# REPRESENTATIONS OF OLD AGE IN THE CONSUMER ELECTRONICS WORLD

**DOI:** 10.1093/geroni/igz038.085

**Published:** 2019-11-08

**Authors:** Mireia Fernández Ardèvol

**Affiliations:** Open University of Catalonia / Universitat Oberta de Catalunya, Barcelona, Catalonia, Spain

## Abstract

This paper examines representations of old age at the Consumer Electronics Show 2019, identifying how explicit product discourses identify later life with Fourth Age dependency, fragility, decline, and care, as is the case with the home and companion-robot industry. While designers take a Fourth Age approach to the ‘senior market’, they liken their products with those for children, as both old and young are stereotyped as requiring surveillance based on their assumed weaknesses. Thus the technological depictions of old age neglect the diversities of older populations and reinforce dominant ageist and homogenizing narratives about older life as disempowering, passive, and digitally divided. Conclusions question why technological design aimed at helping older individuals are uninformed and misconceived about the realities of later life and what recommendations may be offered to resolve this resulting ageism.

